# Effect of short-term peripheral myopic defocus on ocular biometrics using Fresnel “press-on” lenses in humans

**DOI:** 10.1038/s41598-021-02043-2

**Published:** 2021-11-22

**Authors:** Ryo Kubota, Nabin R. Joshi, Inna Samandarova, Maksud Oliva, Arkady Selenow, Amitava Gupta, Steven R. Ali

**Affiliations:** 1 Kubota Pharmaceutical Holdings Co., Ltd., Kasumigaseki Tokyu Building 4F, 3-7-1 Kasumigaseki, Chiyoda-ku, Tokyo Japan 100-0013; 2 Manhattan Vision Associates/Institute for Vision Research, 160 E 56th St. Suite 7, New York, NY 10022 USA

**Keywords:** Neuroscience, Physiology

## Abstract

This study assessed axial length and choroidal thickness changes following short-term peripheral myopic defocus in normal adult subjects. Twenty subjects underwent defocus sessions by viewing a full-field projected movie 4 m away for 4 h in the morning, while wearing spectacle lenses, corrected for distance vision in both eyes. The right eye, serving as the test eye, was peripherally defocused using a Fresnel lens overlay of + 3.50 D with a central clear aperture of 11.5 mm (correlating to a clear central visual field of approximately 23°), while the left eye served as the control (with no Fresnel lens overlay). A subset of 10 subjects from the same cohort also underwent additional defocus sessions with + 5.00 D of peripheral defocus. Axial length was measured and radial sub-foveal choroidal scans were obtained before and after the defocus sessions. The increase in axial length of the test eyes were significantly less than the control eyes under both peripheral defocus conditions (p < 0.05). The difference in mean change for choroidal thickness between test and control eyes was not significant for either dioptric condition. Our results demonstrated that short-term peripheral myopic defocus significantly inhibited axial elongation in adult humans, without significant changes in choroidal thickness.

## Introduction

The human eye is sensitive to the presence of light as well as its optical vergence direction. Several human studies have shown that the eye can sense the direction of full-field defocus relative to the retinal surface, whether myopic, hyperopic, or astigmatic in nature^1–5^. This physiological phenomenon has been investigated by measuring the central axial length (from the anterior part of the cornea to the posterior retinal pigment epithelium) and the central (sub-foveal) choroidal thickness, before and after controlled defocus sessions.

Although the exact mechanism of the biometric changes due to defocus in human eyes remains unclear, it has been shown through experimentation in animal models that the defocus signals are received and interpreted locally within the eye^6–8^. Rymer and Wildsoet proposed a mechanism in which the retina, and/or the retinal pigment epithelium (RPE) can “read” the direction of focus of incoming light rays on the retina and affecting the choroid to actively change its thickness in order to move the retina towards the image plane, via changes in retinal homeostasis mediated by neurotransmitters^9,10^. Hence, in the case of myopic defocus, the relative reduction in axial length would theoretically result from thickening of the sub-foveal choroid; in the case of hyperopic defocus the opposite should occur, in which the increased axial length would result from thinning of the sub-foveal choroid. Additionally, underlying these induced changes in axial length and choroidal thickness, there is also the natural daily circadian rhythm changes in axial length and choroidal thickness^11–14^. In general, during the morning hours the choroid thins while the axial length elongates, and in the afternoon the inverse occurs. Literature states that the diurnal changes of axial length are greater in magnitude than choroidal thickness changes^11–14^. Despite the inverse correlation of axial length and choroidal thickness changes which occur throughout the day, the total variance of change in axial length magnitude cannot be explained *entirely* by the changes in sub-foveal choroidal thickness. This opens up the possibility of other contributing factors such as intra-ocular pressure which also has a rhythmic circadian property^14^.

Read et al., Chiang et al., and Wang et al., all showed that following short term (30 min to 2 h) full-field monocular myopic defocus in humans, the axial length decreased, and the choroidal thickness increased in the test eye. In the control eye, the biometrics underwent normal diurnal changes (i.e., the axial length elongates, and the choroid thins, peaking around noon then possibly reversing in direction)^1,12–16^. Thus, literature clearly shows that the application of full field myopic defocus alters the natural daily variations of axial length and choroidal thickness in human subjects^2,3^.

The effects and possible physiological correlates of peripheral defocus on central biometric components have been explored in various animal models. Smith et al. (2007) showed that peripheral defocus cues only (without central defocus cues) were able to affect axial elongation in rhesus monkeys. They accomplished this by ablating the central 10°–12° of the visual field and then using various plus and minus lenses as defocus stimuli. Their results demonstrated that the retinas responded to form deprivation, and therefore, visual signals from the fovea were not essential for emmetropization and defocus mediated ocular growth^17^. Smith et al. (2020), further showed that the placement of the peripheral defocus zone affects the potency of defocus-mediated ocular growth^18^. After testing different defocus zones, they found that myopic defocus in the near periphery (less than 20° from the fovea) showed the greatest potency in the slowing of axial growth. Contrary to the results obtained in rhesus monkeys, Schippert et al. (2006) failed to show effectiveness of peripheral myopic defocus in guiding a predicted pattern of ocular growth in young chickens^19^. Hence, it can be speculated that the mechanisms and biometrics involved in ocular growth vary across different species. Further research is needed to investigate underlying factors (e.g., duration, magnitude, direction, and location of defocus) that contribute to such physiological changes across different species, especially humans.

Biometric changes following short-term (less than 6 h) peripheral myopic defocus have not been studied in humans. Hence, the objective of our study was to determine whether short-term peripheral myopic defocus would cause predictable changes in central ocular biometry (i.e., axial length shortening and choroidal thickening) in humans. We examined biometric changes in adult human subjects with a non-invasive methodology using two different magnitudes of dioptric (D) defocus supplied by peripherally placed Fresnel lenses, leaving the central 11.5 mm of visual field unaffected (i.e., leaving a clear central visual field of approximately 23°). This diameter was found to be optimal for central visual performance, compared to the other lesser diameters^20^.

## Methods

Twenty (20) subjects with normal vision (13 females and 7 males, mean age 25.5, range = 19–31 years) were recruited for the study. The mean spherical equivalent refractive error of the cohort was − 2.02 D, ranging from plano to − 4.13 D. All the subjects had a refractive cylinder of ≤ 0.75 D. There were 14 Asian subjects and 6 Caucasian subjects. The best-corrected visual acuity was at least 20/20^−3^ in each eye. Exclusion criteria included the following: a history of severe dry eye, a history of strabismus and/or amblyopia, any systemic or infectious conditions or allergies that might interfere with participation, use of systemic or ocular medications known to interfere with vision and/or accommodation, previous ocular surgery or orthokeratology, current myopia control therapy, and current pregnancy or lactation. Written informed consent was obtained from all subjects before enrollment, and the study adhered to the tenets of the Declaration of Helsinki. The Informed Consent Form and protocol were approved by an independent Institutional Review Board (IRB ID # 7716, Sterling IRB, Georgia, USA).

Upon consenting, a complete evaluation of the subjects was performed, which included evaluation of ocular health, visual acuity, and subjective refraction. A non-mydriatic fundus camera Optos Daytona (Optos Incorporated, Marlborough, Massachusetts) was also used to screen for any retinal anomalies. Inter-pupillary distances were measured with a manual pupillometer. The subjects were individually fitted with appropriate frames and CR-39 prescription lenses without any coating, which were best corrected for distance refraction. The subjects then progressed to the defocus study sessions.

The changes in biometric components were explored using a monocular defocus protocol, in which the right eye was chosen as the test eye and the left eye as the control. Similar protocols have been extensively used in the literature to study the physiological effects of full-field monocular defocus stimuli^2,14^. In this study, peripheral myopic defocus was applied with “press-on” Fresnel lenses (3M Health Care, MN, USA) with 11.5 mm central aperture on top of the spectacle lens on the right eye, centered on the pupillary axis. The fully corrected spectacle lens of the left eye had no additional defocus applied. The emmetropic subjects were given spectacles with plano refractive correction, and the Fresnel lenses were positioned accordingly. Two types of metal frames with adjustable nose pads were used. The box measurements of the frames chosen were 55■17, 142 for larger faces and another was 54■18, 137 for smaller face types.

The rationale for the size of the clear, central aperture came from our previous set of experiments where we showed that the 11.5 mm aperture was optimal in terms of visual performance compared to smaller apertures^20^. Two different dioptric powers of the Fresnel lenses were used in the study, + 3.50 and + 5.00 D. Twenty subjects completed the defocus session for the + 3.50 D condition. A subset of 10 subjects from the same cohort also underwent a session with + 5.00 D of peripheral defocus supplied through Fresnel lenses on a different day following at least 24 h of washout period in order to assess a potential effect of a higher dioptric stimulus. Due to operational challenges during the COVID-19 pandemic, the remaining 10 subjects were not able to complete the + 5.00 D defocused condition testing.

### Description of the defocus sessions

The subjects were reminded to be well rested for the day of the experiment as it required their full attention. They were advised to not have any caffeinated or sugary drinks and avoid vigorous exercise during the evening preceding the morning study sessions. The defocus sessions were then started approximately 2 h after their waking time. They were not allowed to have any coffee or sugary drinks during the session. All test sessions started at approximately 8:30 a.m. to assure that the subjects’ eyes were defocused during the ascending phase of the diurnal curve. Before each defocus session, both intra-ocular pressure and blood pressure were measured in all subjects to ensure normal ranges. The illuminance of the room was kept at low photopic levels (30–50 lux) measured with the Sekonic L-858D-U photometer. The subjects were instructed to watch color movies on a screen (8 feet high × 12 feet wide) at 4 m away, while wearing spectacles corrected and fitted for distance refraction (both eyes) for 10 min to “wash out” any effects from the prior near viewing tasks. The screen subtended a visual angle of approximately 42.5° horizontally and 31.5° vertically. Breakfast, low on carbohydrates and sodium, was provided on site. They were separated from their handheld devices such as phones, smart watches, etc. to maintain focus on distance viewing.

**Figure 1:**
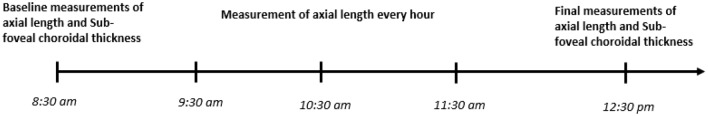
Schematic representation of the measurement procedure milestones.

Subjects during the study complied with maintaining steadiness throughout the defocus sessions and especially during measurements. The subjects sat comfortably in a chair that could be wheeled back and forth between the test instruments, without the subjects needing to stand up and walk towards the instruments themselves. The test instruments were kept in close proximity (of approximately 2 feet away from the subjects). Both the instruments were setup so that when the subjects were resting on the chinrest/headrest arrangement, they were directly facing the screen. During the experiments, the subjects were instructed to be as steady as possible and 15 min prior to measurement time, they were specifically asked to maintain steadiness even more so. The accuracy of the measurements in terms of standard deviation of axial lengths for subjects was approximately 11 microns for the cohort. The measurement of axial length was always performed with glasses off (experimenters removed glasses for the subjects, allowing subjects to sit still). The axial length measurements took approximately 4–5 min per subject and the choroidal thickness measurements took another 4–5 min per subject. Please note that only axial length measurements were performed intermittently during the defocus sessions, and choroidal thickness measurements were only performed before and after the defocus sessions. See Fig. 1 for timeline visualization. Six measurements of axial length for each eye were obtained with a Lenstar LS 900 system (Haag-Streit AG, Koeniz, Switzerland) on 5 different occasions: once before the initiation of defocus (baseline after the “washout”), every hour during the defocus sessions, and at the termination of the session at approximately 12:30 p.m. All the measurements were performed carefully with minimal movement from the subjects as described above under a dimmer illumination of approximately 10–20 lux. The Lenstar LS 900 is a non-contact, optical biometer based on the optical low-coherence principle. We used the automated acquisition mode for all the subjects, which minimized any operator-related biases.

In addition, 2 sets of OCT acquisitions, each consisting of 6 radial spokes under the Enhanced Depth Imaging (EDI) mode, were obtained for each eye in all subjects before and after defocus sessions, with the center of the scan passing through the fovea using a Heidelberg Spectralis OCT2 (Heidelberg Inc., Heidelberg, Germany). The scans were approximately 30° in diameter, and 50 ART scans were obtained with optimum signal to noise ratio. Following an initial quality check, an unmasked designee converted the images into “raw exports” in a “*.vol” format and assigned a random ID to each set of acquisitions taking out any date and time-specific information from the dataset. The OCT exports were then sent to trained, masked researchers to outline the boundary of the chorio-scleral junction using a custom segmentation program developed by the study group that measured the sub-foveal choroidal thickness over the central 0.50 mm. The program first asked the annotators the biometrics of an individual subject’s eye, to account for the longitudinal magnification with respect to a standard schematic eye. The internal limiting membrane (ILM) and Bruch’s membrane (BM) segmentation boundaries were then directly extracted from the Heidelberg data and the annotators outlined the boundary of the chorio-scleral boundaries after correcting for any ILM and BM segmentation errors that were visible. The program also allowed for the annotators to change the brightness and contrast aspect of the images to better segment the boundaries. The segmentation application was verified with a test–retest study performed on 10 random samples yielded a test–retest difference of − 1.26 µm with a standard deviation of 3.3. Because of the subjective nature of chorio-scleral boundary, the inter-subject variations during the annotation procedure were larger than intra-subject variations. Therefore, the same masked annotator was assigned to complete the annotation for a specific subject. This, in practice, reduced variability and increased consistency. The schematic representation of the study measurement timeline is shown in Fig. 1.

For the statistical analysis, the change of axial length and sub-foveal choroidal thickness was determined by comparing the measurements taken at the end of defocus and those taken at baseline. The means and standard error of mean (SEM) were used to describe the distributions, and 2-tailed, unpaired *t* tests were used to evaluate systematic differences (if any). Data was analyzed using Microsoft Excel (Microsoft Corporation, Seattle, WA). GraphPad Prism 8 (GraphPad software, San Diego, CA) was used to perform the analysis and prepare the figures. Trend analysis was also performed with the Prism 8 software and linear fits were obtained to explain the changes seen in control and test eyes under both peripheral defocus conditions. Bonferroni’s correction was applied whenever applicable and a corrected value of p < 0.05 was considered for statistical significance.

## Results

**Figure 2:**
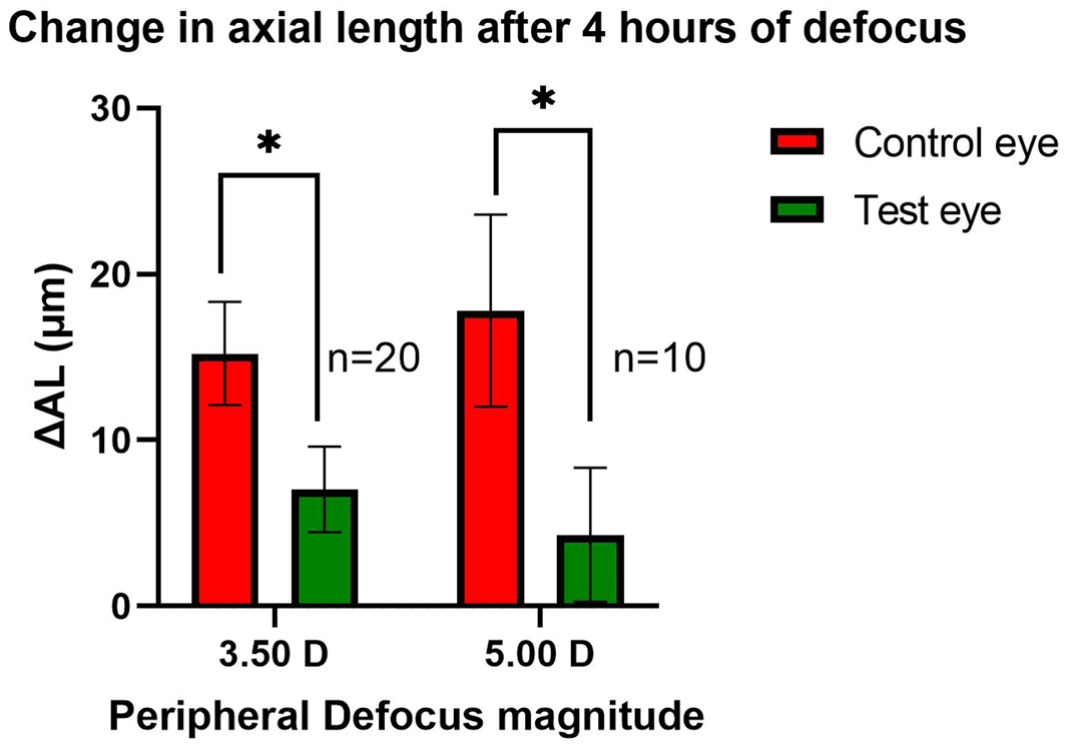
Axial length changes (mean ± SEM) following 4 h of peripheral defocus sessions. Note: *Represent significant differences between test eyes and controlled eyes with corrected p < 0.05.

The mean axial length elongation was significantly less for the test eye as compared to the control eye after 4 h of peripheral myopic defocus for both defocus conditions (t = 2.47, df = 38, corrected p = 0.036 for + 3.50 D defocus condition; and t = 2.73, df = 18, corrected p = 0.027 for + 5.00 D defocus condition after Bonferroni’s correction for multiple comparisons). Additionally, the datasets passed the Shapiro–Wilk test for normality. P values ranged from 0.13 to 0.91. Figure 2 shows the mean axial length change in microns (mean ± SEM) for test and control eyes for both dioptric myopic defocus magnitudes.

**Figure 3:**
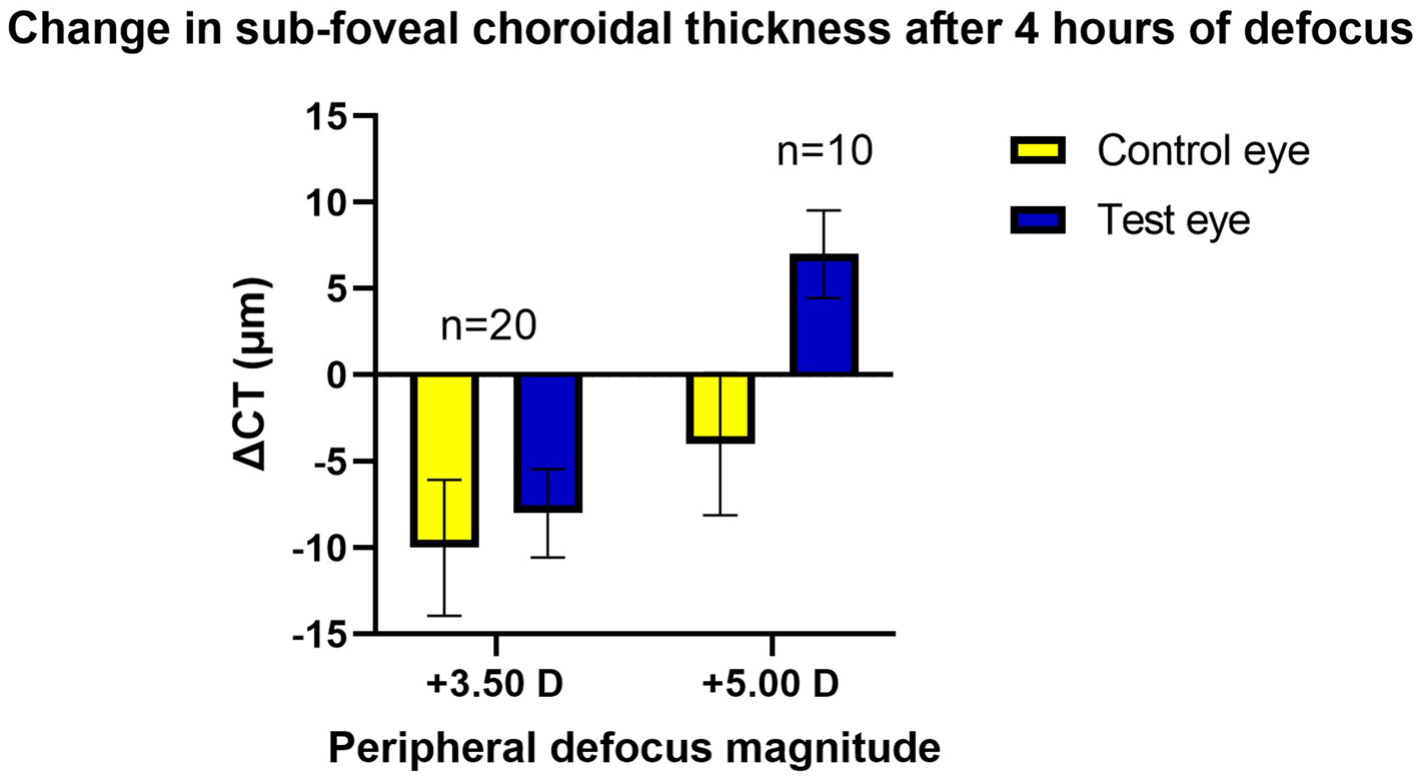
Sub-foveal choroidal thickness changes (mean ± SEM) following 4 h of peripheral defocus sessions.

The magnitude of changes in sub-foveal choroidal thickness were less obvious than those observed for axial length as shown in Fig. 3. For the + 3.50 D peripheral defocus condition, both the test and control eye choroids thinned; however, the magnitude of change was slightly higher for the control eye compared to the test eye (− 10 vs − 8 µm), without statistical significance (t = 0.46, df = 38, at p = 0.64). For the + 5.00 D peripheral defocus condition, the sub-foveal choroidal thickness in the control eye thinned by 4 µm, while the test eye thickened by 7 µm compared to the baseline. The corrected p-value for the differences was still not statistically significant (t = 2.15, df = 18, corrected p = 0.08).

**Figure 4:**
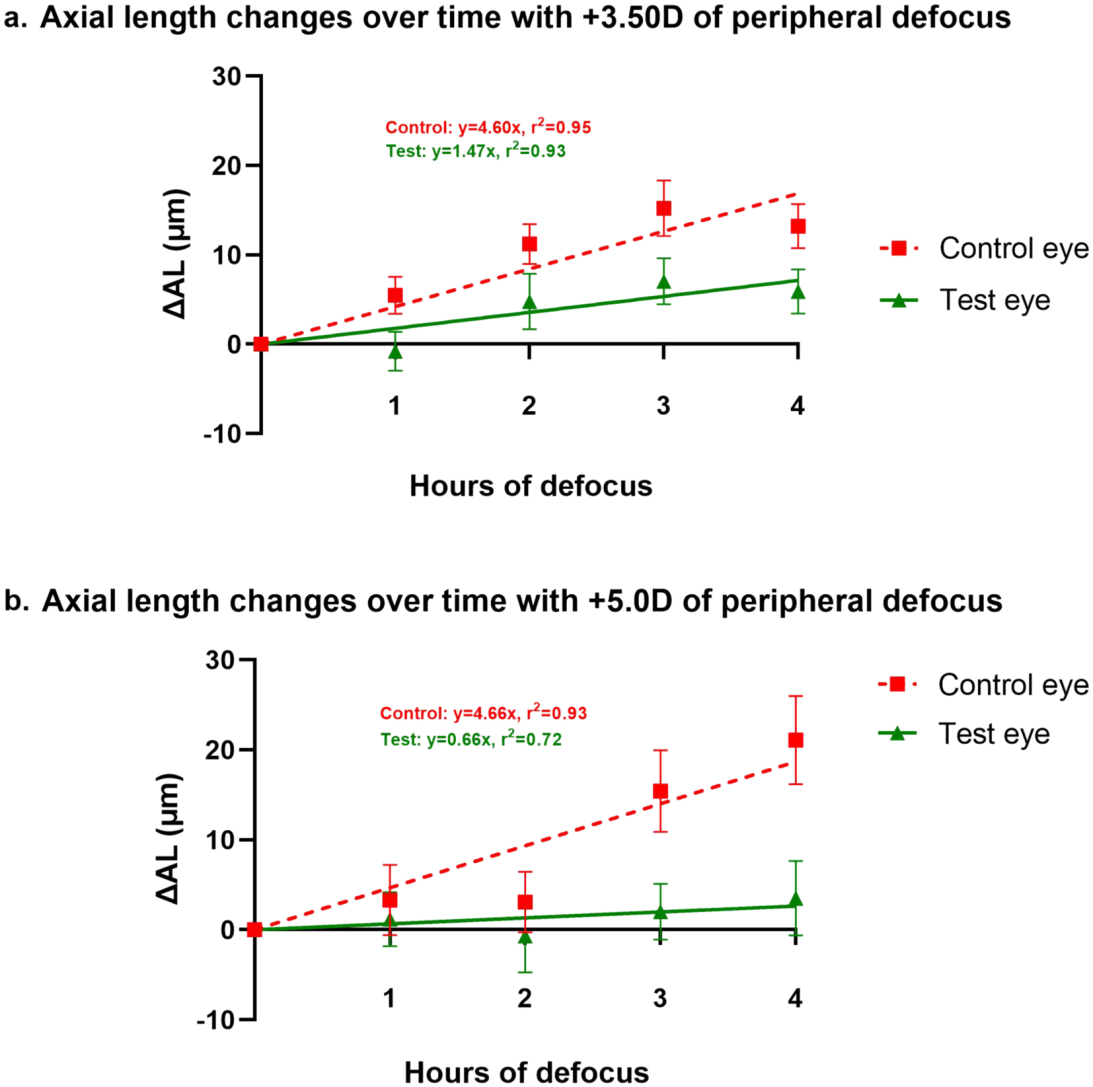
(**a**,**b**) Linear straight line-fits showing mean change in axial length ± SEM in microns over time for + 3.50 D and + 5.00 D of peripheral defocus conditions.

Figure 4 shows the change in axial length of the control eye and test eye over 4 h of defocus (mean ± SE) for the two defocus conditions at each measurement point. Linear regression models were fit with the line passing through the origin (assuming the change is zero at baseline).

As shown in Fig. 4a,b, the rate of increase of axial length for the control eye was larger than that of the test eye.

## Discussion

Pertaining to axial length, we have shown for the first time in human subjects that short-term peripheral myopic defocus alone (with a clear central zone of 11.5 mm diameter) can reduce the elongation of central axial length during the ascending phase of the circadian rhythm. Additionally, this phenomenon was repeatable with two different magnitudes of peripheral defocus i.e., + 3.50 D and + 5.00 D. Furthermore, while not statistically significant, there appears to be a trend in that, the stronger the defocus signal in the periphery, the stronger the tendency of the effect. As shown in Fig. 4a,b, the slope of the linear fit of the test eye results was flatter (i.e., axial elongation was more strongly inhibited) for the + 5.00 D peripheral defocus condition. The effects of peripheral myopic defocus on axial length and choroidal thickness were similar in nature to the effects of full field myopic defocus (as stated in the literature) over time, where the changes in test eyes were consistently smaller than the changes in the control eyes during the corresponding time of the day^2,16^. Thus, our results indicate that the defocus signal processing properties of the human eye remain intact even with only peripheral short-term myopic defocus, resulting in axial length changes along the central axis. Moreover, it can be speculated that when only the peripheral image plane was moved anterior of the retina (i.e., peripheral myopic defocus), the central retina attempted compensation by moving itself forward (i.e., decreasing axial length). Pertaining to changes in sub-foveal choroidal thickness, our results were inconclusive. As shown in Fig. 3, the choroid thinned in the test eyes for + 3.50 D condition, while it thickened for + 5.00 D dioptric condition. Furthermore, the Bonferroni correction rendered all comparisons insignificant.

Read et al. (2010) showed almost a perfect correlation between axial length and choroidal thickness changes following 1 h of full field myopic defocus, i.e., when axial length decreased by approximately 12 µm, the choroidal thickness increased by 12 µm^1^. Similarly, but not so definitively, Wang et al. (2016) found the same opposing trends in axial length and choroidal thickness but without significance for subjects undergoing full field myopic defocus^16^. In our current study, the differences in sub-foveal choroidal thickness changes for test and control eyes following defocus sessions were similar to those reported by Wang et al. (2016). Our results also showed a trend (without reaching statistical significance), whereby the mean choroidal thickness measures for the test eyes were thicker than the control eyes after the defocus sessions.

### Future directions and implications

While this study showed significant findings in the morning hours (i.e., the ascending circadian phase), it would be beneficial to conduct studies in the descending part of the circadian curve to add further insight into the relationship between peripheral defocus and biometric changes. Also, the use of hyperopic defocus and/or light diffusers (i.e., vergence signal diffusers) applied in the periphery can give further insight into the physiological relationship between peripheral image properties and biometric changes.

Finally, from an optical perspective, it can be speculated that when the peripheral image plane was moved anterior to the retina, the central retina attempted compensation by moving itself forward. These findings enhance our understanding of the interaction of biometric aspects of the eye to peripheral myopic defocus. Further research is warranted to understand the underlying mechanisms of ocular biometric changes in response to various peripheral defocus applications whether the application stimuli be short-term versus long-term, myopic versus hyperopic, low versus high dioptric magnitude, central versus peripheral, and small versus large (in size).
